# Editorial: The landscape of the metal complexes: relevant properties and potential applications

**DOI:** 10.3389/fchem.2025.1685795

**Published:** 2025-09-02

**Authors:** Fabio Piccinelli, Albano N. Carneiro Neto, Hermi F. Brito

**Affiliations:** 1 Luminescent Materials Laboratory, Department of Biotechnology, University of Verona, Verona, Italy; 2 Department of Molecular Theory and Spectroscopy, Max-Planck‐Institut für Kohlenforschung, Mülheim an der Ruhr, Germany; 3 Department of Fundamental Chemistry, Institute of Chemistry, University of São Paulo, São Paulo, Brazil

**Keywords:** luminescence, energy transfer (ET), circulalry polarized luminescence, x-ray diffraction, luminescence decay kinetics

The fascinating properties of metal complexes and inorganic materials are increasingly attracting global scientific attention. These compounds play pivotal roles in various research fields, particularly in biomedical applications and materials chemistry. For instance, metal complexes can exhibit antibacterial, antiviral, and antitumor activities. Moreover, the spectroscopic and magnetic properties of both metal complexes and inorganic compounds significantly contribute to technological advancements, including the development of functional materials such as LED phosphors and permanent magnets. Coordination compounds are also crucial for metal ion sequestration and recycling, such as in the recovery of rare earth elements.

This Research Topic focuses especially on the luminescence properties of inorganic-based materials and coordination compounds involving d- and f-block transition metal ions.

In their article, Shyichuk et al. presented a detailed exploration of the mechanisms that govern luminescence evolution in complex systems such as lanthanum fluoride (LaF_3_) doped with Ce^3+^, Gd^3+^, and Eu^3+^ ions. Their approach used a system of rate equations to model the experimental luminescence decay of excited lanthanide states. Notably, in systems with more than three interacting energy levels, the derived decay constants do not map directly to any single process, but instead emerge from the collective behavior described by the rate equations. Multiexponential fitting is essential for capturing such intricate kinetics and drawing meaningful conclusions. In this context, the interplay between the rise and decay phases becomes crucial: a combined rise-and-decay fit can yield significantly more insight than separate analyses. The authors assert that this methodology can reasonably be generalized to other materials and activator species, regardless of their physicochemical nature.

The second contribution, by Al-Tayyem et al. introduced a novel integration of crystallite-specific diffraction probes with *in situ* methods to track the synthesis kinetics of luminescent materials. The authors’ compelling approach involved monitoring ligand-to-metal energy transfer in a representative Tb^3+^-based coordination compound, [Tb(bipy)_2_(NO_3_)_3_] (bipy = 2,2′-bipyridine), using *in situ* luminescence measurements paired with synchrotron X-ray diffraction (XRD). This methodology clarifies the crystallization pathway and reveals the formation of a reaction intermediate. By collecting data at a microfocused synchrotron beamline, a cutting-edge technique that minimizes beam damage and enables crystallite-level interrogation, the study opens exciting avenues for isolating the intermediate and determining its crystal structure in future work. These findings hold immense value for researchers in solid-state chemistry and physics, as they offer new strategies for controlling material synthesis.

Finally, in the study by Poncet et al. the authors directed their attention toward the striking chiroptical properties of Cr^3+^ complexes, which exhibit circularly polarized luminescence (CPL) in the near-infrared (NIR) region, a crucial aspect for biomedical applications. The authors successfully separated the pure PP and MM enantiomers of homoleptic and heteroleptic complexes containing pyridine- and quinoline-based ligands with axial (helical) chirality via chiral stationary phase HPLC. Exceptional CPL activity was recorded in the NIR range (glum values from 0.14 to 0.20 within the 700–780 nm window), achieved through the strategic selection of distinct chiral ligands.

In summary, this Research Topic highlights how innovative approaches and methodologies in the study of optical materials can deepen our understanding of their chemical-physical and photophysical behaviors, along with the key factors that impact their optical performance.

